# Influence of Strain-Offset-Based Yield Definitions on the Accuracy of Finite Element Analysis of 3D-Printed PLA with Different Raster Orientations

**DOI:** 10.3390/polym18020158

**Published:** 2026-01-07

**Authors:** Moiz Majeed, Rafael Silva, Djbril Nd. Faye, Paulo Pedrosa

**Affiliations:** DTx CoLAB, Campus de Azurém, Edifício 1, Universidade do Minho, 4800-058 Guimarães, Portugal

**Keywords:** additive manufacturing, finite element analysis (FEA), nonlinear material modeling, polylactic acid (PLA), strain offset method

## Abstract

Computational mechanics is one of the techniques used to predict and optimize material behavior and structural performance. However, modeling a complex material model and achieving an accurate response in finite element analysis (FEA) remains a challenge. This study investigates the mechanical material properties of 3D-printed polylactic acid (PLA) by integrating tensile testing and FEA to optimize material behavior. The tensile testing was conducted on three different raster orientations (0°, 45°, and 90°), and the resultant stress–strain data were used to calibrate FEA models. For FEA nonlinear material modeling, isotropic elasticity was combined with a multilinear plasticity model, where the yield stress values were determined by using the strain offset method. Six different strain offsets (SOs), i.e., 0%, 0.007%, 0.01%, 0.02%, 0.05%, and 0.2%, were analyzed to evaluate their impact on the accuracy of the FEA results against the experimental results. The results highlight a significant influence of strain offset selection on the plastic region estimation and overall accuracy. The commonly used 0.2% strain offset method (SOM) significantly overestimated the plastic region, while 0% strain offset provided the most accurate simulation response. These results emphasize the importance of selecting the correct yield stress value for 3D-printed nonlinear material modeling in FEA simulations.

## 1. Introduction

Three-dimensional printing is one of the most promising and rapid prototyping technologies that offers great benefits in production cost reduction, material waste, and labor requirements [1]. Initially, 3D printing was only restricted to testing and experimental purposes [2], but, nowadays, due to its ability to manufacture complex geometries and ease of processing, it is widely used in many areas like the aerospace, construction, civil engineering, industrial design, education, medical research, automotive, and robotics industries [3,4,5,6,7,8,9,10,11]. Due to its suitable properties, it has attracted different industries [12].

Polymers are the most commonly used materials in 3D printing and can be processed with various printing technologies. Among these, one of the most promising techniques is the fused deposition method (FDM) [2]. There are many materials available on the market for 3D printing, like polylactic acid (PLA), acrylonitrile butadiene styrene (ABS), thermoplastic polyurethane (TPU), nylon, polycarbonate (PC), polyethylene terephthalate (PET), polypropylene (PP), polyethylene (PE), polycaprolactone (PCL), and polyether ether ketone (PEEK) [13,14,15,16]. Among these, PLA is the most popular due to its properties. It is cheap, easy to print, biodegradable, and stable during the printing process [2,13]. However, the challenge with using 3D-printed PLA is the change in its mechanical properties when compared to bulk PLA due to its dependency on printing parameters, such as infill density, infill orientation, infill pattern, printer nozzle, printer type, bed temperature, printing orientation, and printing speed [1,17,18,19,20,21]. This issue is particularly critical when designing 3D-printed parts for load-bearing applications [22]. Therefore, to optimize its performance, it is important to understand the mechanical properties affecting the materials used for structural load-bearing applications [23].

Tensile testing is a common experimental method used to evaluate key mechanical parameters, such as Young’s modulus, yield stress, ultimate tensile stress, Poisson’s ratio, and elongation strain at break. Several studies can be found in the literature that investigate the tensile properties of 3D-printed PLA [1,2,13,16,20,22,24]. Most of the studies focused on tensile properties because they provide a good understanding of the elastic coefficient of the printed material. It was reported that infill density and raster orientation play a significant role in the tensile strength of the material [25,26,27,28]. It was also reported that choosing different raster angles during 3D printing, such as 0°, 45°, and 90°, has a significant impact on mechanical properties [19]. Furthermore, numerous studies focused on infill density, which is directly proportional to tensile loading and plays a significant role in the strength of 3D-printed materials. In general, a higher infill density results in a higher ultimate tensile strength [29,30]. Therefore, these parameters and material properties can be understood through experiments and computational approaches, which is essential for developing effective material models.

Due to the strong dependency of the manufacturing parameters of 3D-printed PLA on its mechanical behavior, the integration of finite element tools can be a good choice to simulate the structural response of 3D-printed PLA using material properties found through experiments [31]. Other researchers conducted an FEA study to find von Mises stresses, which focused on 3D-printed polymers using 100% infill and comparing the tensile and compression results to better characterize the resulting product [32]. Because of the complexity and high dependency on the currently used procedure, the evaluation of material properties does not produce consistent results that can be used in numerical simulation effectively [2]. Other studies attempted to mechanically simulate 3D-printed parts [33,34]. However, all of them found challenges in obtaining an accurate simulation response that was comparable to the experimental response, which highlights the need for further research on FEA simulation for 3D-printed parts to analyze nonlinear behavior under tensile loading [2,35,36,37,38].

To define nonlinear behavior in FEA, the yield stress calculation method plays a critical role. Most studies have used 0.2% SOM to determine the yield stress from experimental data [2,38,39]. Because of the brittle behavior of PLA, 0.2% SO can overestimate the plastic region, which may lead to wrong simulation responses. Therefore, the influence of the strain offset definition on the accuracy of the FEA of 3D-printed PLA remains unclear. This study addresses this gap by evaluating different strain offset values to understand how the strain offset value can be used to improve the accuracy of the FEA response compared with the experimental response. A similar limitation was reported in finite element studies of cortical bone, which concluded that 0.2% strain offset for yield stress caused the FE model to overpredict force–displacement in the simulation response compared to the experimental response due to the brittle behavior of bone [40].

Therefore, in this study, a nonlinear FEA model was developed to simulate the stress–strain response of 3D-printed PLA to investigate its mechanical behavior under tension. Also, different strain offsets were tested to determine the yield stress values and define their effect on the simulation response. Different infill orientations, i.e., 0°, 45°, and 90°, were tested to ensure and validate the simulation model against different SO yield stress values. The goal was to determine the appropriate yield stress value that accurately represents the experimental data in FE simulation models. Furthermore, the FEA model was validated against experimental data, which was also obtained in this study. This research provides a better understanding for improving FEA models for 3D-printed materials to improve the design and performance of additive manufacturing applications.

## 2. Materials and Methods

### 2.1. Experimental Procedure

**Figure 1:**
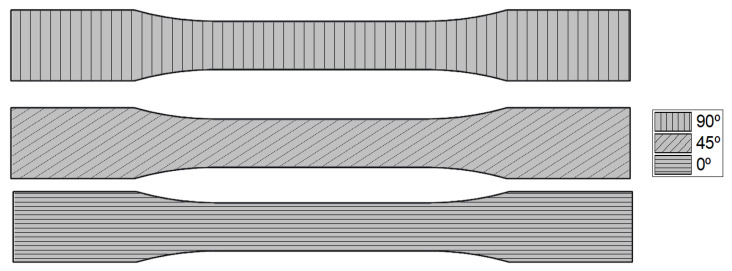
Raster orientations at 0°, 45°, and 90°.

The test specimens using in this work were 3D-printed on a Bambu Lab X1C (Bambu Lab, Shenzhen, China) and the material used was Flashforge PLA filament (Flashforge, Hangzhou, China). Three different raster orientations (0°, 45°, and 90°), representing principal loading cases of load-aligned, diagonal, and transverse printing directions to the applied load [41], were printed as shown in Figure 1. The key process parameters of the 3D-printed specimens are shown in Table 1.

**Figure 2:**
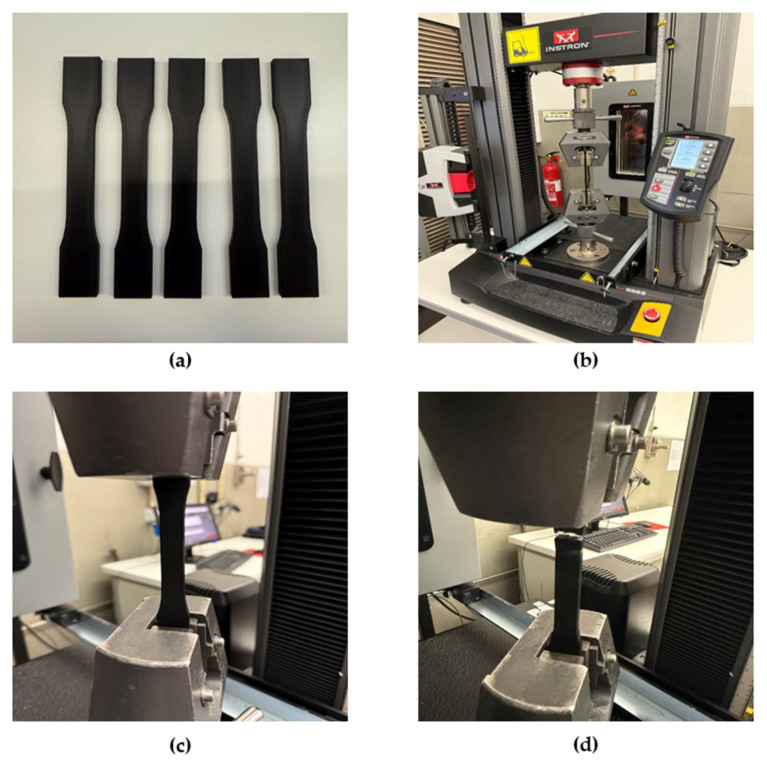
Experimental setup: (**a**) set of five ASTM D638 dog-bone-shaped specimens; (**b**) tensile testing setup; (**c**) specimen under tensile loading; (**d**) failure of the specimen after reaching the breaking point.

**Figure 3:**
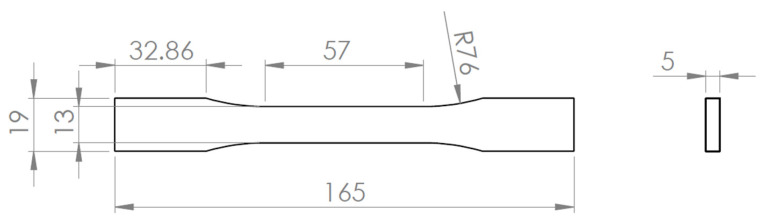
Three-dimensional-printed specimen dimensions, in mm, designed according ASTM D638—Type 1.

Tensile testing was conducted on an Instron 5969 (Instron, Norwood, MA, USA) universal tensile testing machine. Specimens were designed and prepared according to the ASTM D638 standard [42]. To ensure a consistent experimental response, a total of five specimens were printed for each orientation. The tensile testing machine grips were displaced at a speed of 5 mm/min, and the force–displacement data were recorded. The testing setup for the tensile testing can be seen in Figure 2. The specimen geometry, designed using SolidWorks Professional 2025, is shown in Figure 3.

### 2.2. Finite Element Analysis

Regarding the computational technique, FEA was used to simulate the tensile loading on the 3D-printed PLA specimen until ultimate tensile stress, just before necking or failure. Ansys Mechanical Enterprise 2024 R2 was the software used to create the simulations. The same geometry as in the tensile testing experiments was used to capture the similar simulation behavior.

**Figure 4:**
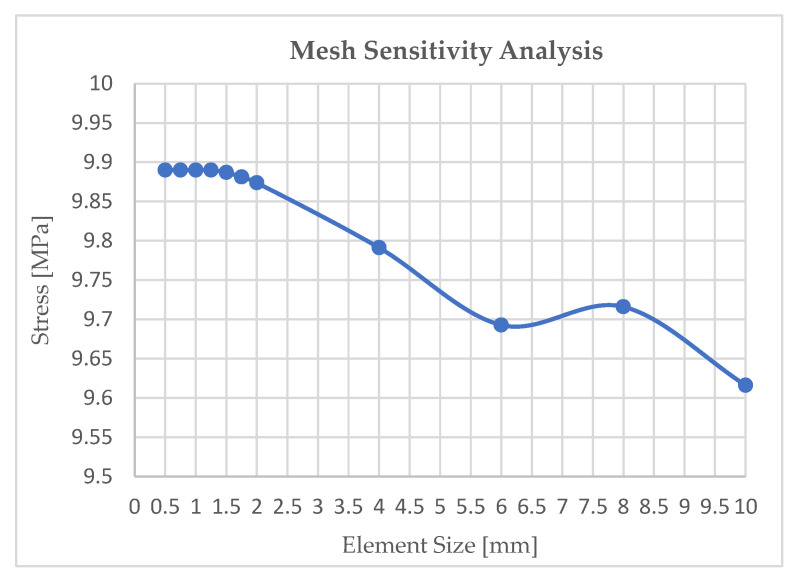
Mesh sensitivity results for FEA setup: maximum von Mises stress for different element sizes.

**Figure 5:**
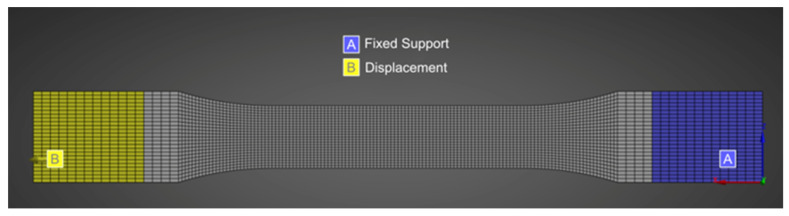
Mesh configuration and boundary conditions used for FEA: applied displacement (yellow) and fixed support (blue).

Figure 4 shows the mesh sensitivity analysis performed to ensure the mesh-independent FEA model produced discrete results only based on boundary conditions, geometry, and material properties. The graph shows that the stress remained unchanged until the element size reached 1 mm, indicating that it did not affect the results and that the results were independent of the mesh. Figure 5 shows the mesh configuration and boundary condition (BC) used in the FEA. Finer mesh was used in the strain gauge area (grey area) compared to the grip area to account for the higher stress concentration in the middle of the specimen while optimizing computational efficiency and ensuring accurate results.

**Figure 6:**
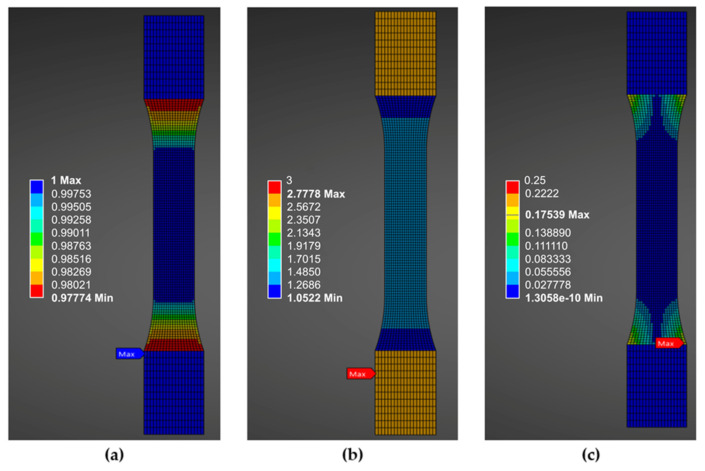
Mesh metrics parameters: (**a**) Jacobian ratio; (**b**) aspect ratio; (**c**) skewness.

For further validation of the mesh quality, mesh metrics were also analyzed in Ansys. As shown in Figure 6, the values of the Jacobian ratio are close to one for all elements, which indicates minimum element distortion and less deviation in simulation results [43]. An aspect ratio of less than three, along with skewness lower than 0.25, ensures solver stability and reliable results.

True stress and true strain were used for FEA because the multilinear isotropic hardening model requires true stress–true plastic strain inputs to accurately represent material behavior beyond the elastic region. Engineering stress is suitable for design-level approximations but does not capture the geometric changes that occur during plastic deformation. Using true stress ensures the correct representation of strain hardening and enables accurate FEA predictions.

#### Material Properties and Yield Stress Calculation

The strain offset method is widely used for brittle and semi-brittle polymers whose stress–strain curves do not display a well-defined yield point. Therefore, the offset method allows a reproducible definition of yield stress, enabling consistent input for FEA material modeling.

For the FEA setup and to accurately capture the material’s nonlinear behavior, isotropic elasticity was combined with a multilinear plasticity model for each infill raster orientation, as shown in Figure 1. To ensure accurate elastic response and initial material stiffness, Young’s modulus (E) was calculated from the linear region of the experimental stress–strain data. However, a Poisson’s ratio (v) of 0.33 was adopted from the literature, as commonly reported for 3D-printed PLA with 100% infill [44,45]. Table 2 summarizes the material properties obtained from experiments for FEA material modeling. The standard deviation values are admissible and correlate well with those in the literature [45].

The raw force–displacement data recorded by the testing machine were converted into engineering and true values using standard relations. Engineering stress ($\sigma_{eng}$) was computed by dividing the applied load by the initial cross-sectional area of the dog-bone geometry. Engineering strain ($\varepsilon_{eng}$) was calculated by dividing the crosshead displacement by the initial gauge length. Although this procedure did not follow the ASTM extensometer requirement, it was applied consistently across all specimens and was suitable for the comparative analysis performed in this study.

To model material plasticity in Ansys, multilinear stress–strain curves were obtained from the experimental data by using Equations (1)–(3) [46,47]. True stress ($\sigma_{true}$) and true strain ($\varepsilon_{true}$) were obtained using the equations based on the assumptions of uniform deformation and volume conservation prior to necking. True plastic strain ($\varepsilon_{plastic}$) was calculated by subtracting the elastic strain component $\sigma_{true}/E$, enabling the extraction of multilinear plasticity curves in Ansys.

(1) $$\sigma_{true}=\sigma_{eng}(1+\varepsilon_{eng})$$

(2) $$\varepsilon_{true}=\ln\left(1+\varepsilon_{eng}\right)$$

(3) $$\varepsilon_{plastic}=\varepsilon_{true}-\frac{\sigma_{true}}{E}$$

where $\sigma_{true}$, $\sigma_{eng}$, $\varepsilon_{eng}$, $\varepsilon_{true}$, $\varepsilon_{plastic}$, and E represent true stress, engineering stress, engineering strain, true strain, true plastic strain, and Young’s modulus, respectively.

The yield stress values were determined using SOM, where different SO percentages, i.e., 0%, 0.007%, 0.01%, 0.02%, 0.05%, and 0.2%, were analyzed to find the most suitable offset that optimized the simulation response compared to the experimental response. During the material modeling, it was noticed that the yield stress significantly influence the simulation response. To improve accuracy, different SO values were evaluated to obtain a close correlation between the FEA response and experimental response.

## 3. Results and Discussions

This section presents and analyzes the experimental and simulation results. A comparison between the results is conducted by evaluating different SO values to determine the most accurate correlation.

### 3.1. Tensile Testing Results

**Figure 7:**
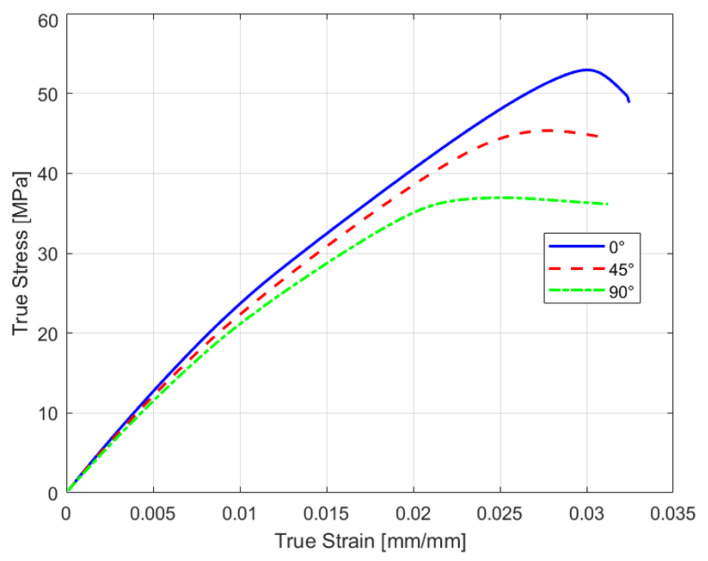
True stress–strain representative curve for different raster orientations: 0°, 45°, and 90°.

A total of five specimens were tested at each raster orientation angle. Figure 7 presents the true stress–strain representative curves for each raster orientation, as true stress-strain data are required in Ansys Workbench 2022 R2 for accurate simulation setup [46].

The curve shows that the 0° raster orientation produced the strongest specimen with an ultimate tensile stress (UTS) of 52.93 MPa. The 45° raster orientation produced the second strongest specimen, and 90° was the weakest (considering the specific manufacturing parameters shown in Table 1).

### 3.2. FEA Simulation Results

**Figure 8:**
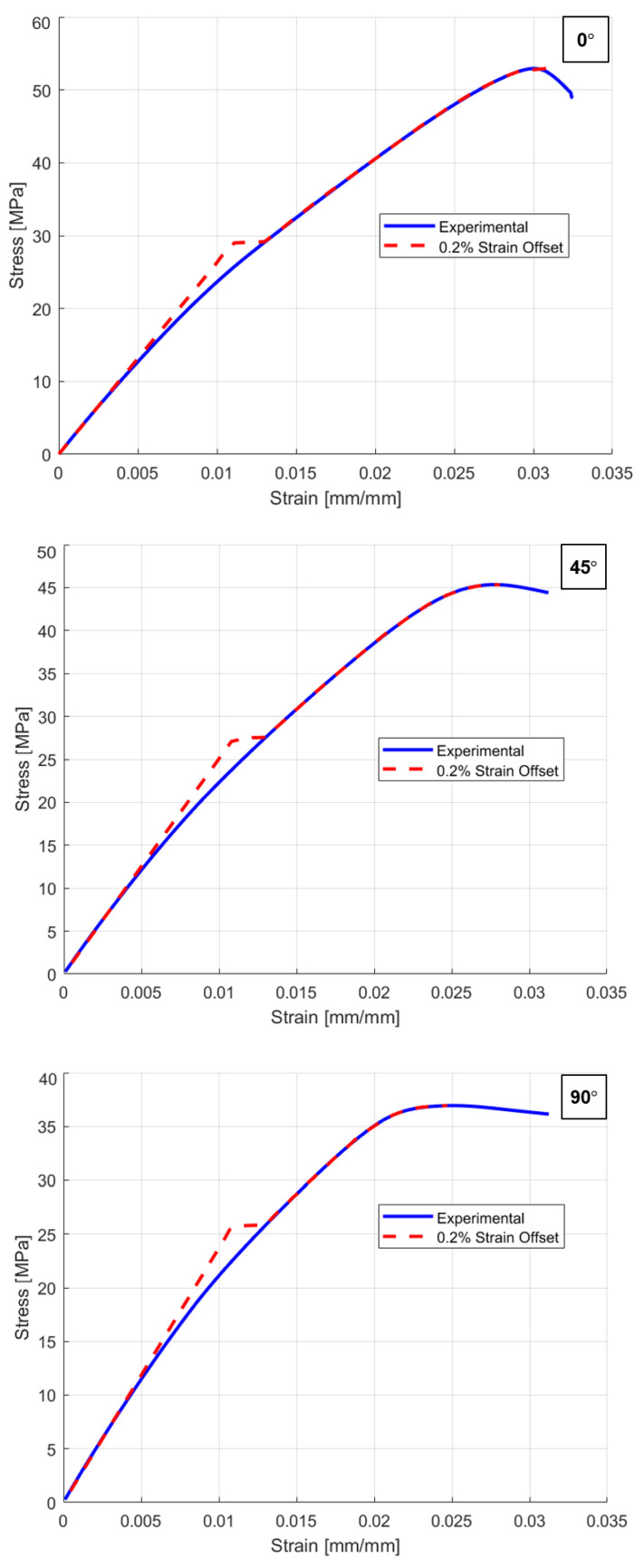
Comparison between experimental and simulation responses with 0.2% SO yield stress for 0°, 45°, and 90° raster orientations.

To investigate the impact of SO selection on the yield stresses among the raster orientations, an FEA model was developed. Additionally, as previously discussed in Section 2.2, a mesh sensitivity test was conducted, and mesh metrics were evaluated to ensure that the simulation results were independent of element size. The conventional 0.2% strain offset method [39,40], which is commonly used to find yield stress values, was initially applied for material modeling for all raster orientations. Figure 8 shows a comparison between the experimental and simulation results, where 0.2% SO yield stress overestimates the plastic region, resulting in simulation responses being inconsistent w experimental responses with approximate absolute relative errors (AREs) of 5.84%, 5.75%, and 5.46%, respectively.

**Figure 9:**
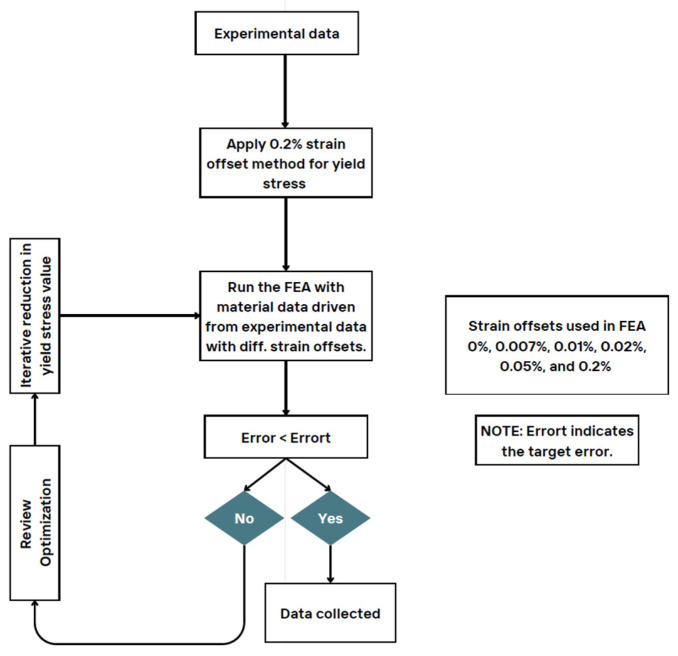
Optimization flow chart.

**Figure 10:**
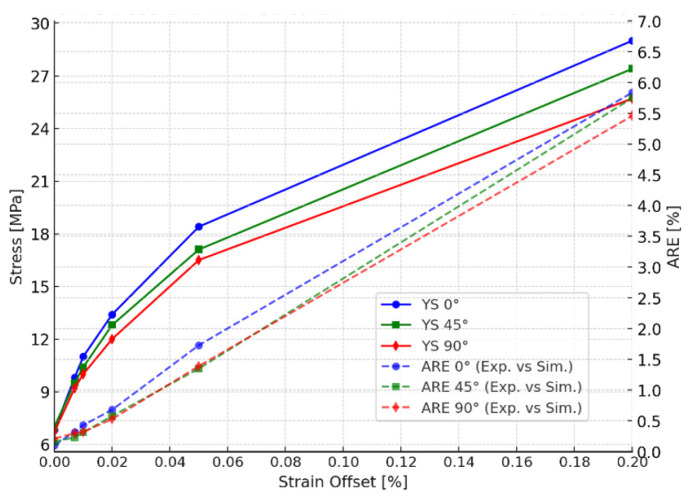
Yield stress values and absolute relative error (ARE) values calculated for different strain offsets and raster orientations.

To optimize the material model and achieve less absolute relative error (ARE) between simulations and experimental responses, the optimization methodology presented in Figure 9 was applied. Apart from 0.2% SO, lower SOs of 0%, 0.007%, 0.01%, 0.02%, and 0.05% were tested. Figure 10 presents the ARE of the simulation response compared to the experimental response, alongside the yield stress values for different strain offset percentages. The comparison between the experimental and simulation results for various strain offset points reveals that a 0.2% strain offset leads to an overestimation of the plastic region. However, reducing the offset values results in improved consistency between the simulation and experimental responses.

### 3.3. Validation and Discussion

Three key findings can be highlighted among the results of this study. First, the strain offset selected to define the yield stress value has a significant impact on the accuracy of FEA, with 0% strain offset providing the simulation response closest to the experimental response. Second, raster orientation has a strong influence on the mechanical response of 3D-printed PLA, confirming its anisotropic behavior. Third, isotropic material modeling was used to approximate the experimental response for each rater orientation; further improvement may be achieved through material modeling.

The results obtained in Figure 10 highlight the importance of selecting an accurate yield stress value to perform precise FEA simulations. The results show that the conventional 0.2% SOM may not always provide accurate material representation, especially for 3D-printed materials like PLA. Reducing the strain offset values also reduces the ARE, which demonstrates that the ARE and SO values are directly proportional to each other in the current case scenario. Table 3 presents data corresponding to Figure 10, which shows a comparison between the yield stress values for different strain offsets and AREs (%) between experimental and simulation responses.

**Figure 11:**
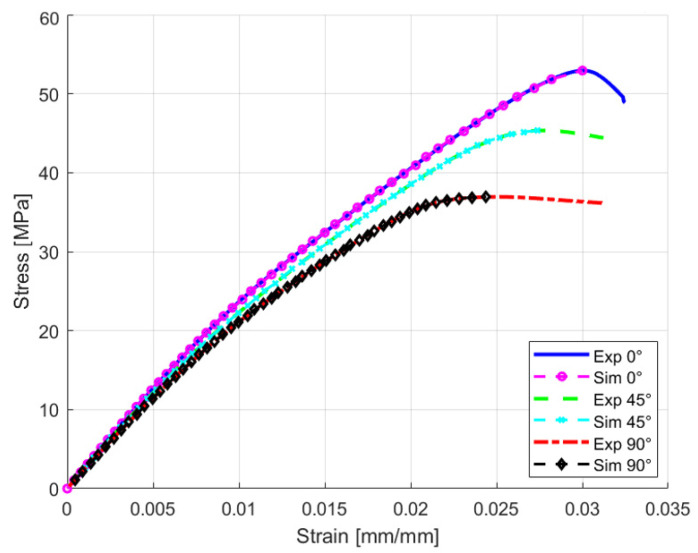
Comparison between optimized simulation responses against experimental responses by using 0% strain offset for 0°, 45°, and 90° raster orientations.

Table 3 shows that 0% SO provides the best results, with AREs of 0.1%, 0.16%, and 0.21% for 0°, 45°, and 90° raster orientations, respectively. Notably, by using 0% SO, the yield stress is very low compared to that found in the other studies in the literature [2,13,16,48]. However, it accurately reflects the specific manufacturing parameters used during 3D printing [12,29,49]. Table 4 summarizes the final findings, including the optimized material properties extracted using the 0% strain offset method for FEA material modeling. Additionally, Figure 11 shows a comparison between the optimized simulation response and the experimental response, demonstrating the improved accuracy achieved with this approach. Due to the brittle nature of the material, a similar limitation of the traditional 0.2% strain offset method has been documented in finite element studies of cortical bone, where it resulted in an overprediction of force–displacement responses [36]. This analogy supports the methodology of reducing the strain offset used for materials exhibiting limited plastic deformation, even though bone and PLA are different materials.

**Figure 12:**
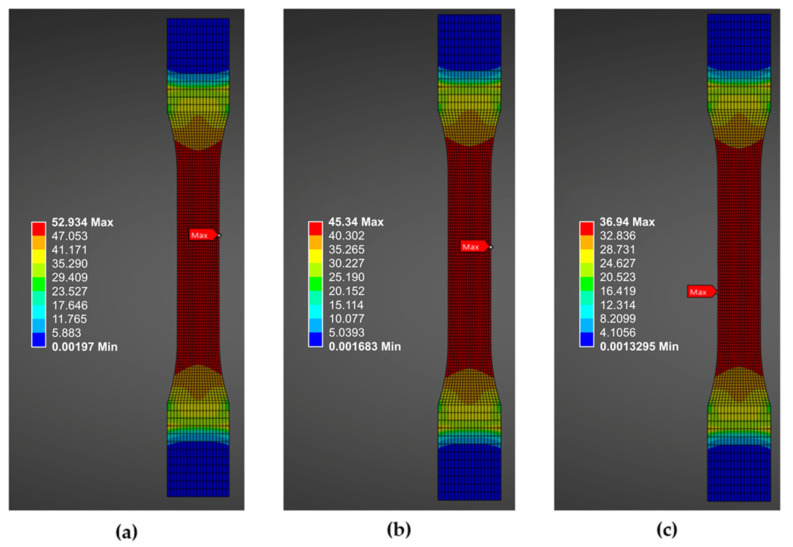
von Mises stress distribution for different raster orientations: 0°, 45°, and 90° are presented in (**a**–**c**), respectively.

Since this study did not focus on the fracture behavior of PLA, Figure 12 presents the von Mises stress distributions for 0°, 45°, and 90° raster orientations, highlighting the maximum stress region before failure. These stress distributions can help predict potential crack initiation points and serve as for validating simulation models via comparison with the experimental maximum stress values listed in Table 4.

## 4. Conclusions

This study underlines that selecting the appropriate yield stress value to define the plasticity model in FEA plays a significant role in the accuracy of the simulation results. Under all raster orientations, the conventional 0.2% strain offset consistently overestimated the plastic region, resulting in larger discrepancies between numerical and experimental responses. Based on our findings, SOM with an offset of 0% is recommended for calculating the yield stress in tensile tests with specific manufacturing parameters. In addition, the selection of the strain offset value is strongly dependent on the manufacturing parameters (such as raster orientation and infill structure) as well as the deformation measurement method used during tensile testing. Consequently, the strain offset should not be assumed universally but rather optimized for each specific printing setup to obtain reliable FEA material models.

As shown in this work, PLA exhibits orientation-dependent mechanical properties, including variations in Young’s modulus and ultimate tensile strength, under different raster orientations (0°, 45°, and 90°). This study highlights that while an isotropic material model can approximate the response for each raster orientation, considering the anisotropic material model leads to more realistic results. However, this study only focuses on the isotropic material model to understand the fundamental material behavior based on manufacturing parameters and working conditions. The isotropic material approach can be used to predict material properties while optimizing computational efficiency. Therefore, the isotropic material approach must be used before developing a more complex model such as anisotropic models.

Future work may include anisotropic modeling and/or different test types such as three-point bending tests; different materials like PC, PET, or TPU; and different 3D-printing parameters and techniques (SLA). Additionally, different strain rates can be explored to investigate the material’s responses and the influence of different SO% values on simulation responses.

## Figures and Tables

**Table 1:** Three-dimensional printing parameters used for specimen production.

Processing Parameter	Value
Nozzle diameter	0.4 mm
Layer height	0.2 mm
Layer width	0.4 mm
Infill pattern	Aligned rectilinear
Infill density	100%
Nozzle temperature	220 °C
Building temperature	55 °C
Printing speed	~250 mm/s

**Table 2:** Material properties of 3D-printed PLA used in FEA for different raster orientations (0°, 45°, and 90°).

Properties	0° Raster Orientation	45° Raster Orientation	90° Raster Orientation
E [MPa]	2629.30 ± 34.86	2507.10 ± 63.12	2368.9 ± 10.20
UTS [MPa]	52.93 ± 0.90	45.33 ± 0.37	36.94 ± 0.50

**Table 3:** Data corresponding to Figure 10. Yield stress values and absolute relative error (ARE) values calculated for different strain offsets and raster orientations.

Raster Orientation	Strain Offset %	Yield Stress [MPa]	ARE % exp. vs. sim.
0°	0	6.8	0.10
	0.007	9.8	0.32
	0.01	11	0.43
	0.02	13.4	0.68
	0.05	18.4	1.73
	0.2	29	5.84
45°	0	7	0.16
	0.007	9.5	0.23
	0.01	10.4	0.31
	0.02	12.8	0.58
	0.05	17.1	1.35
	0.2	27.4	5.75
90°	0	6.7	0.21
	0.007	9.2	0.30
	0.01	10	0.32
	0.02	12	0.53
	0.05	16.5	1.39
	0.2	25.7	5.46

**Table 4:** Optimized material properties at 0% SO for 0°, 45°, and 90° raster orientations.

Properties	0° Raster Orientation	45° Raster Orientation	90° Raster Orientation
E [MPa]	2629.30	2507.10	2368.9
YS [MPa]	6.8	7	6.7
UTS [MPa]	52.93	45.33	36.94

## Data Availability

The raw data supporting the conclusions of this article will be made available by the authors on request.
